# Plum-blossom needle plus Chinese herbal medicine for alopecia areata: A protocol for systematic review and meta analysis

**DOI:** 10.1097/MD.0000000000022515

**Published:** 2020-10-09

**Authors:** Genhua Tang, Jun Xiong, Qian Fan, Han Guo, Xingchen Zhou, Siyuan Zhu, Zhiying Zhong, Jun Chen, Lunbin Lu

**Affiliations:** a Jiangxi University of Traditional Chinese Medicine, Nanchang, PR China; b Affiliated Hospital of Jiangxi University of Traditional Chinese Medicine, Nanchang, PR China; c Changshu Hospital of Traditional Chinese Medicine, Changshu, PR China.

**Keywords:** alopecia areata, Chinese herbal medicine, Plum-blossom needle, protocol, systematic review

## Abstract

**Background:**

Alopecia areata (AA) is a common, inflammatory, nonscarring type of hair loss that is characterized by depression, anxiety and social isolation. In recent years, Plum-blossom needle plus Chinese herbal medicine has gradually shown its clinical advantages and been more and more widely used in China. Whereas, there has been no systematic review and meta-analysis. The purpose of this study is to estimate the safety and effectiveness of Plum-blossom needle plus Chinese herbal medicine in AA treatment.

**Methods:**

Seven databases as following: PubMed, Embase, Cochrane Library, China National Knowledge Infrastructure (CNKI), Chinese Scientific Journal Database (VIP), and Chinese Biomedical Literatures Database (CBM) will be searched from their inception to August 2020. Two reviewers (LBL and ZYZ) will respectively regulate research selection, data extraction, and risk of bias assessment. A third reviewer will be settled to consulting, if necessary. Review Manager Software 5.4 will be implemented for this study.

**Results:**

The results will be published in a peer-reviewed medical journal. This meta-analysis will provide a synthetic review of the credible evidence for the treatment of Plum-blossom needle plus Chinese herbal medicine with AA.

**Conclusions:**

This systematic review and meta-analysis expects to provide high-quality evidence regarding the synergistic effect of Plum-blossom needle plus Chinese herbal medicine treatment for AA.

## 1 Introduction

### 1.1 Description of the condition

Alopecia areata (AA) is an extremely common autoimmune condition affecting hair characterized by patches of nonscarring alopecia affecting scalp and body hair that can be psychologically exterminating.^[1]^ Despite multidisciplinary endeavour, its aetiology is not entirely known, and AA is natural history is unpredictable, although some evidence suggests that genetic factors, immunological and environmental could be generating the disease.^[2]^

At present, the global incidence of AA is 2% of the population suffering from the situation during alopecia areata patients’ lifetime, the majority of AA patients starting before the age of 30 years. However, the prevalence of AA can vary between 0.1% and 6.9% depending on the population researched. In China, AA prevalence approximately.27% of the population presents this disease^[3]^; In the United Kingdom, 2% of its population; In the United States, AA affects between 0.7% and 3% of the individual; In Mexico, the reported affects between 0.2% and 3.8%; In Korea, it has been estimated that AA prevalence between 0.9% and 6.9% of the individuals.^[1]^ At the same time, AA does not distinguish men or women, although some study suggest a slight inferior in men.^[4]^ While AA affects both sexes equally, data from the Rochester Epidemiology Project showed that men tended to be diagnosed earlier compared with women (mean age at diagnosis, 31.5 vs 36.2 years).^[5]^

Many theories have been developed regarding the etiological factors of AA, which contain the involvement of an toxic or infectious factors, neuropathic and endocrine disturbance theories, genetic factors and autoimmunity.^[6]^ The Major theory of AA pathogenesis is that it is an autoimmune appearance during to a break in hair follicle immune privilege. The nosogenesis of AA is an issue in dispute.^[7]^

AA is hard to treatment, and regrettably, no widespread completely recognized treatment exists for all situations. Nevertheless, British and Japanese treatment guidelines for AA have recommended partial immunotherapy as one of the most efficient choices. Intralesional, local or systemic corticosteroids are administered to patients with AA. There are many option include anthralin, cyclosporine, sulfasalazine, adalimumab, etc.^[8]^ Treatments are selected according to the individuation of patients, which are oriented towards eliminating inflammation, controlling symptom sand preventing hair loss, but followed by some adverse consequences. Some drugs are causing adverse effects such as avascular necrosis, weight gain, hypertension, sleep disturbances, diabetes, mood changes, atypical hair coloration acne, sensitivity to allergies or even diseases such as vitiligo.^[9]^ Therefore, a large number of clinical studies have been put into effect onthistopic to reduce the clinical symptoms of AA with efficiency and safety. Nevertheless, no systematic reviews have reported the effectiveness of Plum-blossom needle plus Chinese herbal medicine on AA. Thus, we expect to conducted this protocol of moxibustion as an intervention for AA patients in order to achieve a better treatment effect.

### 1.2 Description of the intervention

Acupuncture is a unique effective traditional therapeutics with a history of three thousand years. The treatment can protect neurons and promotes axonal regeneration to treat miscellaneous disorders.^[10]^ Plum-blossom needle is one of the acupuncture therapy which is an significant treatment method in traditional Chinese medicine (TCM) that has the preponderances of convenience, high tolerance, low cost, and minimal adverse reactions. Chinese herbal medicine (CHM), has been integrated as an important part of healthcare in China.^[11]^ It has been commonly used for AA. Plum-blossom needle plus Chinese herbal medicine has been evidenced to be a well-tolerated and effective therapy for the treatment of skin diseases.

TCM is a effective prospecting treatment which is increasingly being accepted around the world as a therapeutic choice for conducting various physical conditions. Plum-blossom needle plus Chinese herbal medicine may be gain an advantage over topical immunotherapy with respect to alleviating depressive symptoms and the extent of hair loss in patients with AA.

### 1.3 How the intervention might work

The theory underlying the therapeutic effect of Plum-blossom needle plus Chinese herbal medicine on AA remains ill-defined. The apply of combined acupuncture and Chinese herbal medicine therapy possibly reduce the severity of hair loss play a positive role in AA.

### 1.4 Why is it necessary to perform this study?

Combined Plum-blossom needle and Chinese herbal medicine are commonly apply to AA treatment. However, there is no meta-analysis or systematic review appraised evidence for their synergistic treatment effect.^[12]^ To evaluate this synergy based on original research, a systematic review could yield allows the evaluation of the effectiveness and safety of each therapy and the highest level of evidence. Therefore, this study is essential.

### 1.5 Objectives

The purpose of this systematic review and meta-analysis is to evaluate the effectiveness of Plum-blossom needle plus Chinese herbal medicine in treating AA and provide reliable recommendations.

## 2 Methods

### 2.1 Study registration

This study is registered on the International Prospective Register of Systematic Reviews in INPLASY, registration number: INPLASY202080038 (https://inplasy.com/inplasy-2020-8-0038/) on 9 August, 2020. This report will be conducted based on the preferred reporting items for systematic reviews and meta-analyses protocols (PRISMA) statement guidelines.^[13]^

### 2.2 Inclusion criteria

#### 2.2.1 Study type

All randomized controlled trials (RCT) study on Plum-blossom needle plus Chinese herbal medicine therapy treatment of alopecia areata. Others such as case reports, animal experiments, non-RCTs, or RCT protocol will be exclude.

#### 2.2.2 Participants

We will consider patients with a validated diagnosed criteria of AA irrespective of their gender, age, severity, education, and disease duration.

#### 2.2.3 Type of intervention

Trials will included Plum-blossom needle plus Chinese herbal medicine therapy with intervention. While sole treatment will be excluded, for example, Chinese herb decoction, warm-needling, fire needling, and other sole treatment. The control group should receive selective Western medicine or Chinese herbal medicine treatment.

#### 2.2.4 Type of outcome measures

##### 2.2.4.1 Primary outcomes

Primary outcome measures will include:

1. The effective rate.
2. Rate of hair loss.
3. Response rate.

##### 2.2.4.2 Secondary outcomes

Primary outcome measures will include:

1. Quality of life.
2. Satisfaction with the appearance of hair.
3. Incidence of any adverse events (AEs).

### 2.3 Exclusion criteria

The exclusion certain contain the following items:

1. The literature related to the same study, and duplicated publications
2. Unable to get literature for available data or full text through various means

### 2.4 Search strategy

The following electronic databases will be searched: PubMed, The Cochrane Library, EMBASE, Chinese Biomedical Literature Database (CBM), China National Knowledge Infrastructure (CNKI), Technology Journal Database and China Science, and Wan Fang Databases (WF). We will think about studies announced between the database initiation and September 2020. Similar search strategies will be used for all electronic databases. We will also manually search eligible trial, which is unpublished or ongoing. The search strategy for PubMed is provided in Table 1.

**Table 1:**
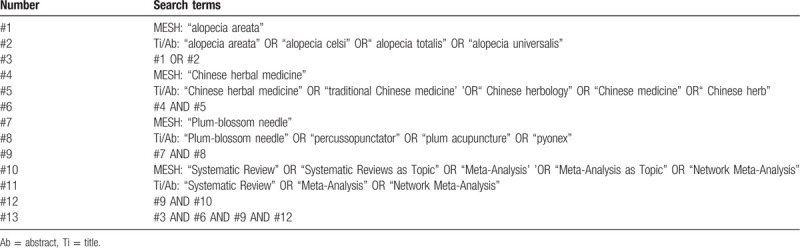
Search strategy for the PubMed database.

### 2.5 Study selection

**Figure 1:**
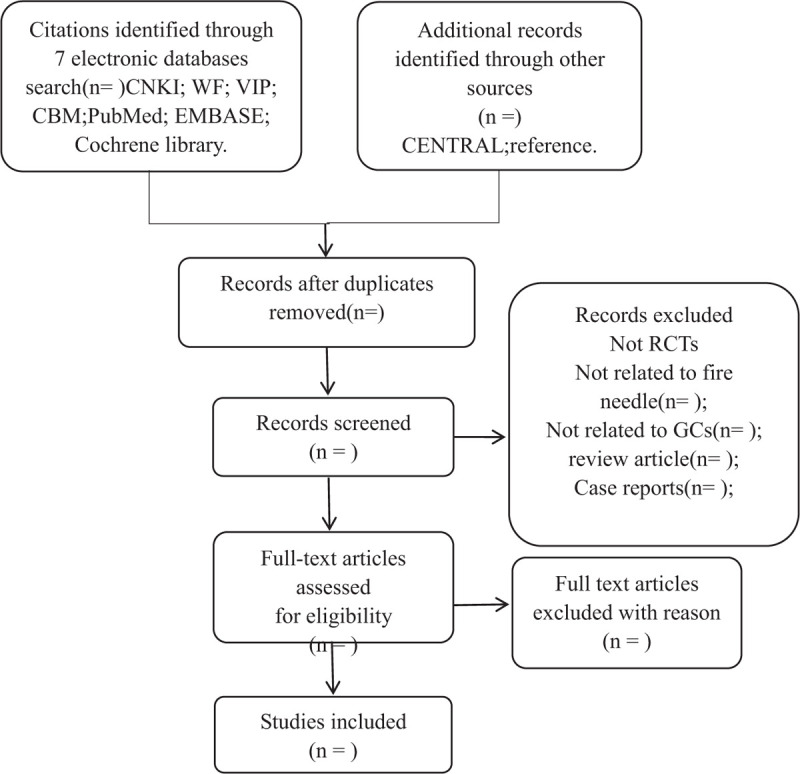
Flowchart of literature selection.

One reviewer will seek for potentially related studies. Subsequently, the retrieved studies will be imported into NoteExpress3.2.0 for duplicate counting and removal. NoteExpress3.2.0 software will delete the duplicate document from all the obtained studies. Next, 2 reviewers will read the full text to determine the final included studies and separately critique all the qualified studies. Titles and abstracts will be scanned to exclude unrelated records. Subsequently, 2 reviewers will read the full text for further filtration where disagreements will be made by consultation with the third researcher. The above process will be presented as a PRISMA flowchart (Fig. 1).

### 2.6 Data extraction and management

1. Two independent reviewers (GHT and HG) will use NoteExpress3.2.0.7535 and Excel software 11.1.0.9912 to extract data. The extracted information will be saved in electronic form.
2. Review authors will independently screen the titles and abstracts of records obtained by searching the electronic databases to determine potential eligibility. Data extraction and cross-check will be managed afterwards independently. Any discrepancies regarding study selection will be resolved through discussion and reach an agreement, and if necessary, arbitrated by the third reviewers. In this step, we will use NoteExpress3.2.0.7535.
3. The research team designed structured data extracted information including: the first author, nationality, publication year, patients’ necessary information, descriptions of studies, sample size, interventions of both the test group and controlled quality, group, randomization, allocation concealment, primary outcomes, secondary outcome measures, etc.

### 2.7 Risk of bias assessment

Two reviewers (SYZ and JC) will separately assess risk-of-bias from the guidelines of Cochrane Reviewer's Handbook 5.0.24.^[14]^ This tool in included studies will be evaluated according the following domains: blinding of participants, allocation concealment, random sequence generation, blinding of participants and outcome assessors selective reporting, incomplete outcome data, and other bias. Each of them will be classified into “low,” “unclear,” or “high” based on information provided by the trials. The evaluated domains will be separately evaluated by 2 reviews, and differentials will be addressed by consulting a third reviewer.

### 2.8 Data synthesis and analysis

#### 2.8.1 Data synthesis

Data synthesis will be completed using RevMan5.4 software. Measurement of the treatment effect. Ninety-five percent confidence intervals (95% CI) and risk ratios (RR) will be used to dichotomous variables, and weight mean differences (WMD) with 95% CI will be used to continuous variables.

#### 2.8.2 Heterogeneity assessment

We will appraise among-study heterogeneity according to the guidelines of RevMan5.4 Software. The value of I^2^ will be used for heterogeneity calculations. The random-effect and mixed-effect models will be adopted, if I^2^ ≥ 50% (significant heterogeneity) and <50% (minor heterogeneity), respectively. When the results are substantial heterogeneity or considerable heterogeneity, subgroup analysis and sensitivity analysis will be made to look for the possible causes.

#### 2.8.3 Data synthesis

If studies are adequately homogeneous in design and comparison, we will conduct all statistical analyses will be implemented using Review Manager Software 5.4. The fixed-effects model or random-effects model will be chosen according to the I^2^ value. A 95% CI will be the effective size for data synthesis. We will perform a narrative, qualitative summary if the data is not fit for quantitative analysis.

#### 2.8.4 Subgroup analysis

If the necessary data are available, we will carry out subgroup analysis according to the following topics: the type of AA, period of treatment, type of Plum-blossom needle plus Chinese herbal medicine and the type of intervention in the control group or the study group.

#### 2.8.5 Sensitivity analysis

A sensitivity analysis will be conducted when there is important heterogeneity according to the following aspects: heterogeneity qualities, sample size, characteristic of studies, and methodological element. If heterogeneity is reduced after small and low-quality studies are excluded, and we must be more careful in concluding.

#### 2.8.6 Reporting bias

If enough trials (≥10 trials) are included, the funnel plots will be used to assess publication bias. Otherwise, we will perform the Egger test with STATA 13.0 Software.

#### 2.8.7 Grading the quality of evidence

We will use the Grading of Recommendations Assessment, Development, and Evaluation (GRADE) to assess the quality of evidence, the main outcomes including the five aspects (study limitations, inconsistency, imprecision, indirectness, and publication bias).^[15]^ The quality of evidence will be rated as four levels as following: high, moderate, low or very low, which according to the five aspects (inconsistency, limitations, imprecision, indirectness, and publication bias).

#### 2.8.8 Ethics and dissemination

Given that this is a systematic review of the effectiveness of Plum-blossom needle plus Chinese herbal medicine in patients with AA, it does not involve individual data. Therefore, ethical approval is not required. Upon completion of analyses, the results will be reported in a peer-reviewed journal.

## 3 Discussion

AA is a disease that has a great influence on patients’ psychology, resulting in reduced self-esteem and negatively affect the quality of life. The internationally recommended treatment is topical immunotherapy for extensive disease and intralesional corticosteroids for localised hair loss in patches. But topical immunotherapy is not always effective in treating the disease. RCTs have proved that Plum-blossom needle plus Chinese herbal medicine is effective in treating AA with little side effects. Therefore, we hope that this study can provide a high level of evidence-based evidence for the effectiveness and safety of Plum-blossom needle plus Chinese herbal medicine in the treatment of AA, and guide clinical decision-making.

## Author contributions

**Conceptualization:** Genhua Tang, Jun Xiong.

**Data curation:** Han Guo, Siyuan Zhu, Zhiying Zhong.

**Formal analysis:** Xingchen Zhou, Lunbin Lu.

**Investigation:** Jun Xiong, Zhiying Zhong, Lunbin Lu.

**Methodology:** Genhua Tang, Qian Fan, Jun Chen.

**Software:** Han Guo, Xingchen Zhou.

**Supervision:** Jun Xiong, Han Guo.

**Writing – original draft:** Genhua Tang, Jun Xiong, Siyuan Zhu, Zhiying Zhong.

**Writing – review & editing:** Qian Fan, Jun Chen.

## Abbreviations

Abbreviations: AEs = adverse events, CI = confidence interval, GRADE = Grading of Recommendations, Assessment, Development, and Evaluation, PRISMA = preferred reporting items for systematic reviews and meta-analyses, RCTs = randomized controlled trials, RR = risk ratios, TCM = traditional Chinese medicine, WMD = weight mean differences.
